# The Impact of Strength/Resistance Training on Related Outcomes of Patients with Knee Osteoarthritis: A Systematic Review and Meta-Analysis Study: Strength/Resistance Training in Knee Osteoarthritis

**DOI:** 10.31661/gmj.v14i.3720

**Published:** 2025-04-16

**Authors:** Farzaneh Yazdi, Amirali Salajegheh, Fatemeh Yazdi Yahyaabadi, Pouria Salajegheh

**Affiliations:** ^1^ Neuroscience Research Center, Institute of Neuropharmacology, Kerman University of Medical Sciences, Kerman, Iran; ^2^ Department of Pharmaceutical Sciences, Tehran University of Medical Sciences, Tehran, Iran; ^3^ Department of Pediatrics, Kerman University of Medical Sciences, Kerman, Iran; ^4^ Department of Pediatrics Hematology and Oncology, Endocrinology and Metabolism Research Center, Kerman University of Medical Science, Kerman, Iran

**Keywords:** Knee Osteoarthritis, WOMAC, KOOS, VAS, Resistance Training, Stiffness, Outcome

## Abstract

**Background:**

The aim of this study was to systematically evaluate and synthesize the available evidence on the effects of resistance/strength training on physical function, stiffness, pain, and quality of life of patients with knee osteoarthritis (KOA).

**Materials and Methods:**

A systematic search of electronic databases, including PubMed, Scopus, WOS, and the Cochrane Library, was performed to identify trials published up to December 2024 evaluating the effects of resistance/strength training on KOA. Eligible studies included interventions targeting pain, stiffness, physical function, or quality of life, compared to control or alternative exercise groups. Pooled effect sizes were calculated using fixed and random effects models based on the degree of heterogeneity observed among the studies, with the random effects model applied due to significant variability in outcomes.

**Results:**

A total of 21 randomized controlled trials, including 2,345 participants, were analyzed. Resistance training significantly improved physical function (MD=−3.02, P0.01) and reduced stiffness (MD=−0.46; P=0.03). Pain outcomes showed mixed results, with significant reductions observed on the WOMAC pain scale (MD=−0.83, P=0.04), but not consistent effects across other measures such as the VAS.

**Conclusion:**

Resistance training improves physical function and stiffness in KOA, with mixed effects on pain and no significant impact on quality of life. High heterogeneity highlights the need for standardized protocols. Resistance training is a valuable component of knee OA management.

## Introduction

Knee osteoarthritis (KOA) is one of the most prevalent degenerative joint disorders globally, disproportionately affecting older adults and individuals with a history of joint injury, obesity, or repetitive strain on the knees. Characterized by the progressive degradation of articular cartilage, joint inflammation, pain, stiffness, and restricted range of motion, knee KOA poses significant challenges to patients’ quality of life [1][2]. It is a leading cause of disability, with global estimates suggesting that millions of individuals experience its debilitating effects daily. As the aging population grows and sedentary lifestyles become more common, the burden of knee KOA is projected to increase, making effective management strategies a critical priority for healthcare systems worldwide [3][4].The management of knee KOA typically focuses on symptom alleviation and functional improvement, encompassing a combination of pharmacological, non-pharmacological, and surgical approaches. Pharmacological treatments, including nonsteroidal anti-inflammatory drugs (NSAIDs) and intra-articular injections, offer symptomatic relief but are often associated with potential adverse effects, particularly with long-term use. Similarly, while surgical options such as total knee arthroplasty can provide substantial benefits for advanced KOA, they are invasive, costly, and carry risks of complications [5][6]. These limitations highlight the importance of non-pharmacological interventions as a cornerstone of knee KOA management, particularly in the early and intermediate stages of the disease. Exercise-based interventions have garnered widespread attention for their role in managing musculoskeletal disorders, including knee KOA. Physical activity is widely regarded as a safe and effective method to improve joint function, alleviate pain, and enhance overall quality of life [7][8][9]. Among various exercise modalities, strength and resistance training stand out due to their targeted approach in addressing the biomechanical and neuromuscular deficiencies associated with knee KOA. Resistance training focuses on enhancing the strength of the muscles surrounding the knee joint, particularly the quadriceps, which play a pivotal role in joint stabilization and load distribution. Improved muscle strength has been shown to reduce joint stress, enhance functional capacity, and potentially slow disease progression [10][11][12].

Despite the promising benefits, the evidence surrounding the effectiveness of strength and resistance training for knee KOA is heterogeneous. While some studies report significant improvements in pain relief, physical function, and muscle strength, others highlight inconsistencies in outcomes, potentially due to variations in intervention protocols, patient characteristics, and methodological quality. These discrepancies underscore the need for a comprehensive synthesis of the available evidence to establish clear guidelines for the implementation of resistance training in knee KOA management [13][14][15][16][17][18][19][20][21][22]. Moreover, there remains a lack of consensus on the optimal parameters of resistance training, such as intensity, frequency, duration, and exercise type, for maximizing therapeutic benefits in individuals with knee KOA. The interaction between resistance training and other factors, such as comorbidities, age, and severity of KOA, also warrants further investigation. A robust synthesis of existing evidence can address these gaps and provide actionable insights for clinicians and researchers. Hence, this systematic review and meta-analysis aim to evaluate the impact of strength and resistance training on key outcomes in patients with knee KOA, including pain, physical function, and quality of life.

## Materials and Methods

**Figure-1:**
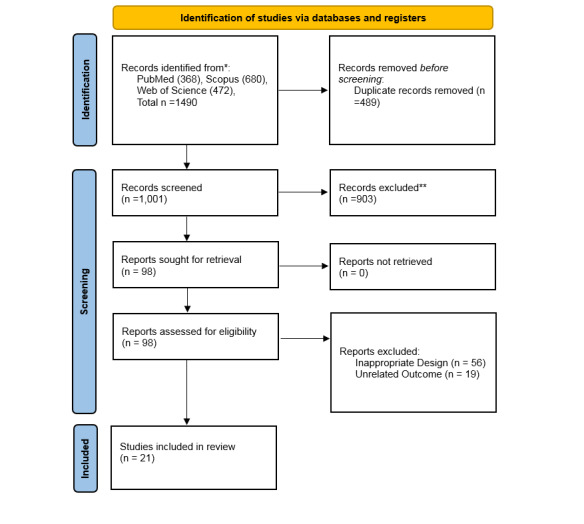
Figure 1. The PRISMA flowchart diagram of the included studies

**Table:** Table1. Characteristics of the Included Studies

**First Author**	**Year**	**Design**	**N**	**Control Group**	**Age**
*Topp et al.* *[23] *	2002	RCT	102	Yes	60-65
*Foley et al.* *[24] *	2003	RCT	105	Yes	70.9
*Jan et al.* *[25] *	2008	RCT	98	Yes	62
*Lund et al.* *[26] *	2008	RCT	79	Yes	65-70
*Lim et al.* *[27] *	2010	RCT	66	Yes	65
*Jorge et al.* *[28] *	2015	RCT	60	Yes	60
*Cheung et al.* *[29] *	2017	RCT	83	Yes	72
*Gomiero et al.* *[30] *	2017	RCT	64	Yes	61.6
*Alfieri et al.* *[31] *	2020	RCT	39	Yes	63.7
*Assar et al.* *[32] *	2020	RCT	36	Yes	55-63
*Holm et al.* *[33] *	2020	RCT	90	Yes	63-66
*Skoffer et al.* *[35] *	2020	RCT	59	Yes	70
*Hsu et al.* *[36] *	2021	RCT	42	Yes	65
*Messier et al.* *[34] *	2021	RCT	377	Yes	65
*Onwunzo et al.* *[37] *	2021	RCT	40	Yes	58
*Rafiq et al. (i)* *[38] *	2021	RCT	50	Yes	53.12
*Rafiq et al. (ii)* *[39] *	2021	RCT	56	Yes	54.6
*Kus et al.* *[42] *	2022	RCT	48	Yes	58
*Osama et al.* *[40] *	2022	RCT	24	Yes	57
*Joshi et al.* *[41] *	2023	RCT	54	Yes	55-58
*Oistad et al.* *[43] *	2024	RCT	168	Yes	57

This systematic review and meta-analysis were conducted following the Preferred Reporting Items for Systematic Reviews and Meta-Analyses (PRISMA) guidelines. The aim of this study was to assess the efficacy of resistance training (RT) in the treatment of knee osteoarthritis (KOA). The methodology was designed to ensure a comprehensive and unbiased synthesis of available evidence.

### Systematic Search

A comprehensive literature search was performed across multiple databases, including PubMed, Cochrane Library, Embase, Web of Science, and Scopus. The search included all available records up to August 2024. Relevant Medical Subject Headings (MeSH) and keywords were used, specifically focusing on terms such as "knee osteoarthritis," "resistance training," "strength training," and "exercise training." Additional sources were identified by manually screening the reference lists of relevant articles and previous systematic reviews to ensure that no pertinent studies were overlooked. Pooled effect sizes were calculated using fixed and random effects models based on the degree of heterogeneity observed among the studies, with the random effects model applied due to significant variability in outcomes.

### Inclusion and Eligibility

The eligibility criteria for this study were defined according to the PICO framework. The Population (P) included clinical studies on human patients diagnosed with RA based on the American College of Rheumatology (ACR) criteria. The Intervention (I) was low-level laser therapy (LLLT), while the Comparison (C) involved placebo, sham treatment, or standard care. The primary Outcomes (O) of interest were the mean differences (MD) in pain relief, joint stiffness, physical function, and inflammatory markers. Studies were excluded if they were non-randomized, involved animal models, were case reports, or lacked clear clinical outcomes or sufficient data for extraction. Additionally, studies focusing on other types of arthritis or conditions were excluded.

### Data Extraction and Outcome Measures

Data extraction was independently conducted by two reviewers using a standardized data collection form. Extracted data included study characteristics (e.g., author, publication year, country), patient demographics (e.g., age, gender, disease duration), details of the RT protocols, control conditions, and outcome measures (e.g., mean differences in pain, joint stiffness, physical function in WOMAC, VAS, and KOOS indices). Discrepancies between the reviewers were resolved through discussion or with the involvement of a third reviewer if necessary.

### Statistical Analysis and Data Synthesis

The pooled mean differences (MD) in outcomes between the resistance training (RT) and control groups were calculated using a random-effects model to account for potential heterogeneity among studies. The analysis was conducted using RevMan software (version 5.4, The Cochrane Collaboration City of publication: Copenhagen, Denmark). The I² statistic was employed to assess the degree of heterogeneity across the included studies, with thresholds of 25%, 50%, and 75% representing low, moderate, and high heterogeneity, respectively.

In cases where the I² value exceeded 50%, a random-effects model was consistently applied to account for the variability among studies. Conversely, if the I² value was 50% or lower, a fixed-effects model was utilized. The Mantel-Haenszel method was used to pool effect sizes, and standard deviations were calculated for continuous outcomes. A z-test was conducted to evaluate the overall significance of the pooled effect sizes and to compare the significance between subgroups.

Subgroup Analyses and Sensitivity Analyses: Subgroup analyses and sensitivity analyses were not applicable in this study due to the limited number of included trials and the homogeneity of the study populations. As a result, the analysis focused on the overall effects of resistance training without further stratification. Publication bias was assessed by visually inspecting funnel plots, and any asymmetries were further evaluated using Egger’s test. All statistical analyses, as well as the creation of forest and funnel plots, were performed using R (R Foundation for Statistical Computing, Vienna, Austria), supplemented by RStudio (RStudio Inc., Boston, MA). (Specifically, the "metafor" package in R was utilized for conducting meta-analyses and generating visualizations, providing advanced statistical capabilities and flexibility in data handling).

Quality Assessment: The methodological quality of the included studies was assessed using the Cochrane Risk of Bias Tool. Each study was evaluated for potential biases in areas such as randomization, blinding, and incomplete outcome data. Additionally, the overall quality of evidence was graded using the GRADE approach, which considers factors such as study design, risk of bias, inconsistency, indirectness, imprecision, and publication bias. This assessment revealed that while many studies demonstrated a low risk of bias, some exhibited limitations that may affect the reliability of the findings.

## Results

**Figure-2:**
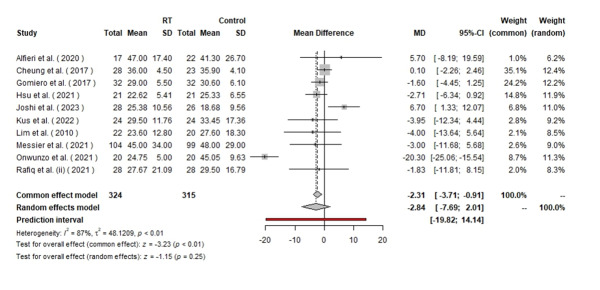
Figure 2. Forest plot of the overall WOMAC

**Figure-3:**
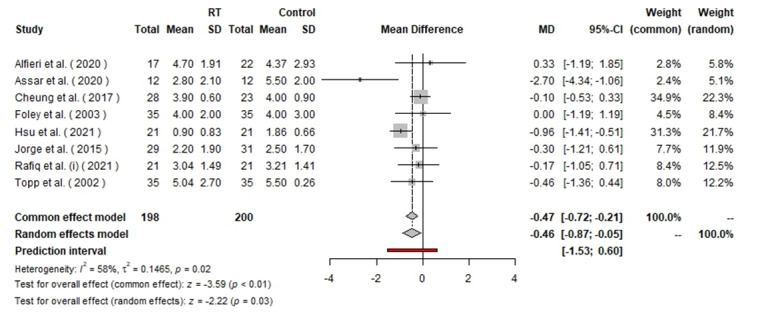
Figure 3. Forest plot of the stiffness subscale of WOMAC

**Figure-4:**
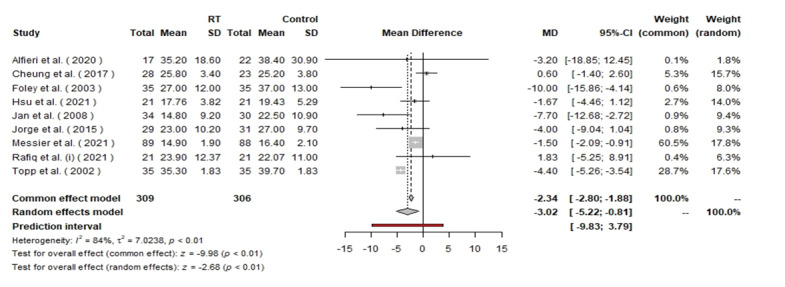
Figure 4. Forest plot of the function subscale of WOMAC

**Figure-5:**
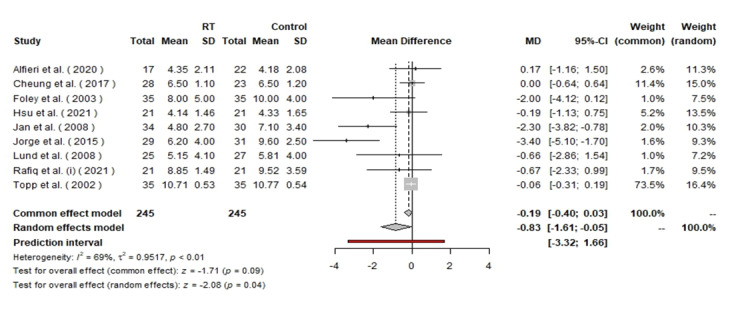
Figure 5. Forest plot of the pain subscale of WOMAC

**Figure-6:**
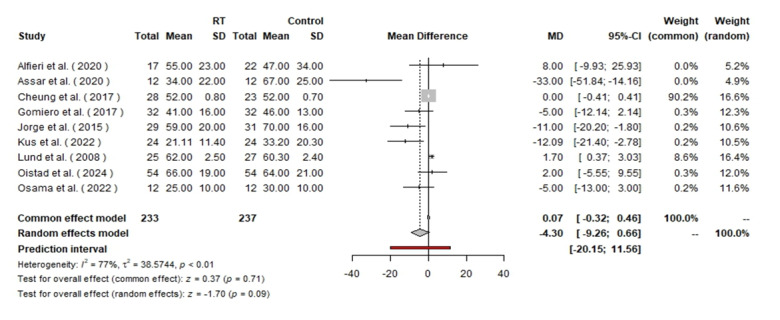
Figure 6. Forest plot of the VAS for pain

**Figure-7:**
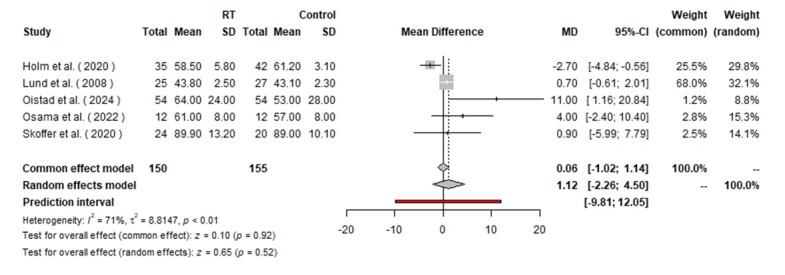
Figure 7. Forest plot of the pain subscale of KOOS

**Figure-8:**
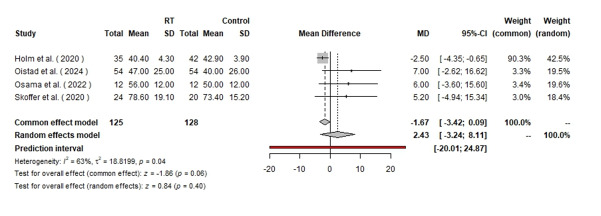
Figure 8. Forest plot of the quality-of-life subscale of WOMAC

Our initial search across PubMed, Scopus, and Web of Science resulted in 1490 articles. After removing 489 duplicates, 1,001 unique records remained. We then screened the titles and abstracts of these records, leading to the retrieval of 98 full-text articles for further assessment. Following a thorough evaluation, 21 studies met the inclusion criteria and were incorporated into the systematic review [23][24][25][26][27][28][29][30][31][32][33][34][35][36][37][38][39][40][41][42][43], with 21 of these studies also included in the meta-analysis. The detailed characteristics of the included studies are summarized in Table-1 and Figure-1.

### WOMAC (Western Ontario and McMaster Universities Osteoarthritis Index)

In the analysis of the overall WOMAC scores, a total of 639 patients were included, with 324 patients in the resistance training (RT) group and 315 in the control group. Given the reported high level of heterogeneity (I²=100%), only the results from the random effects model are presented to avoid misleading interpretations. The mean difference (MD) under the random effects model was −2.84, with a 95% confidence interval (CI) of [−7.69, 2.01]. This result suggests a trend favoring resistance training, though it is not statistically significant (P=0.25). The substantial heterogeneity indicates high variability among the included studies, which may be attributed to differences in study populations, intervention protocols, and outcome measures (Figure-2). For the WOMAC stiffness subscale, the pooled analysis included 398 patients (198 in the RT group and 200 in the control group). The random effects model yielded an MD of −0.46 with a 95% CI of [−0.87, −0.05], suggesting a statistically significant reduction in stiffness favoring the RT group (P=0.03). The heterogeneity was moderate, with I²=58% and P=0.02 (Figure-3). The analysis of the WOMAC function subscale included a total of 615 patients, with 309 in the RT group and 306 in the control group. Under the random effects model, the MD was −3.02 with a 95% CI of [−5.22, −0.81], indicating a significant improvement in function for the RT group (P<0.01). Heterogeneity was high at I²=84%, P<0.01 (Figure-4). In the WOMAC pain subscale analysis, a total of 490 patients were evaluated (245 in each group). The random effects model showed an MD of −0.83 with a 95% CI of [−1.61, −0.05], indicating a statistically significant reduction in pain with RT (P=0.04). The heterogeneity in this analysis was also high, which further supports the decision to report only random effects results (Figure-5).

Given the high level of heterogeneity observed across the studies, the source of this variability was not formally investigated in this analysis due to the limited number of studies and the complexity of potential confounding factors. Future research should aim to explore these sources of heterogeneity, as understanding the underlying differences in study populations, intervention characteristics, and outcome measures could provide valuable insights into the effectiveness of resistance training for knee osteoarthritis.

### VAS (Visual Analog Scale)

The VAS pain analysis included 470 patients, with 233 in the RT group and 237 in the control group. The random effects model showed an MD of −4.30 with a 95% CI of [−9.26, 0.66], indicating a trend toward pain reduction with RT, though not statistically significant (P=0.09). The fixed effect model produced an MD of 0.07 with a 95% CI of [−0.32, 0.46], showing no significant difference between groups (Figure-6).

### KOOS (Knee Injury and Osteoarthritis Outcome Score)

In the analysis of KOOS pain scores (Figure-7), 305 patients were included (150 in the RT group and 155 in the control group). The random effects model yielded an MD of 1.12 with a 95% CI of [−2.26, 4.50], indicating no significant effect of RT on pain reduction according to this scale (P 0.52). The fixed effect model produced an MD of 0.06 with a 95% CI of [−1.02, 1.14] similarly showing no significant effect (P=0.92).

Finally, in the analysis of KOOS quality of life, 253 patients were analyzed, with 125 in the RT group and 128 in the control group. The random effects model yielded an MD of 2.43 with a 95% CI of [−3.24, 8.11], which was not statistically significant (P=0.4). The fixed effect model showed an MD of −1.67 with a 95% CI of [−3.42, 0.09], also indicating no significant effect (P=0.06). The heterogeneity was moderate, with I2=63% (Figure-8). Further information regarding the analysis of the publication bias is available in Appendix 1 to 7.

## Discussion

This systematic review and meta-analysis evaluated the effects of resistance training on several key outcomes in patients with knee osteoarthritis, including overall pain, stiffness, functional capacity, and quality of life. The results showed that resistance training was associated with improvements in certain outcomes, specifically reducing WOMAC stiffness and improving WOMAC function scores. The random effects model demonstrated statistically significant reductions in stiffness (MD=−0.46, P=0.03) and functional impairment (MD=−3.02, P<0.01). However, findings for overall pain, as measured by the WOMAC pain subscale and VAS, were less consistent. While there was a trend toward pain reduction, particularly in the WOMAC pain subscale (MD=−0.83, P=0.04), some analyses, including KOOS pain scores, did not show significant differences. Furthermore, no significant improvements were observed in the quality of life as assessed by the KOOS QoL subscale.

High heterogeneity across analyses indicates that study-level factors may influence the observed effects of resistance training, suggesting the need for further investigation into factors such as intervention duration, intensity, and patient characteristics.The findings of this meta-analysis are consistent with previous research suggesting that resistance training can have beneficial effects on physical function and stiffness in individuals with knee osteoarthritis. Prior studies have demonstrated that resistance training may alleviate symptoms by strengthening the muscles surrounding the knee joint, particularly the quadriceps, which helps stabilize the joint and reduce mechanical stress on the affected area. A meta-analysis by Fransen et al. (2015) similarly found significant improvements in physical function and stiffness with exercise interventions, including resistance training, in knee OA populations, supporting the results of our analysis for the WOMAC stiffness and function subscales [44]. However, our findings diverge somewhat in terms of pain reduction, with mixed results across different pain measures. Some prior studies, such as Lange et al. (2008), reported consistent pain relief following resistance training, while others found more variable effects, particularly when comparing pain reduction on specific scales like the VAS versus WOMAC pain scores [45][46][47][48]. Additionally, while some studies reported quality-of-life improvements, our analysis did not find significant effects in this domain, which may be attributed to the short duration or limited intensity of resistance training in some included studies. A recent study reported significant improvements in functional mobility and reduced stiffness following a 12-week resistance training program tailored for knee OA patients, echoing the reductions in WOMAC stiffness and function scores observed in our analysis [27][49][50]. Similarly, another study demonstrated that moderate-intensity resistance training led to improvements in both joint stability and perceived stiffness among older adults with knee OA, reinforcing the positive functional outcomes observed in our study [42][51].

However, recent studies present mixed findings regarding pain relief, which is consistent with the variable pain outcomes in our meta-analysis. For example, a large-scale randomized controlled trial conducted found that while resistance training significantly improved functional outcomes, its effects on pain, measured via both VAS and KOOS pain scales, were modest and not consistently statistically significant [43][52]. These findings suggest that while resistance training may contribute to pain reduction, it may not be sufficient as a standalone intervention for pain management in knee OA. Regarding quality of life, our findings are consistent with those of recent studies that report limited improvements in this area [19][53]. A study found that while patients experienced functional benefits from resistance training, quality-of-life improvements, as measured by the KOOS QoL subscale, were minimal. This may be due to the fact that quality of life in knee OA patients is influenced by multiple factors beyond physical symptoms, such as mental health, social participation, and overall disease progression. Therefore, while resistance training positively affects physical outcomes, additional interventions addressing psychosocial factors might be necessary to achieve meaningful changes in quality of life [22][54]. For clinicians, implementing resistance training protocols tailored to the needs of knee OA patients can be a practical approach to symptom management. Exercise programs should ideally focus on the quadriceps and other muscles supporting the knee to enhance joint stability and function. Low- to moderate-intensity resistance training appears to be beneficial, as recent studies indicate that such levels of intensity are generally safe and effective for older adults, who are the primary population affected by knee OA [55][56][57]. Clinicians may consider incorporating resistance training as part of a multimodal treatment plan, particularly for patients who are not candidates for or prefer to avoid pharmacological interventions due to potential side effects.

Several limitations must be acknowledged. High heterogeneity across studies, particularly for pain and quality of life, indicates variability in study populations, intervention protocols, and outcome measures, complicating the interpretation of pooled results. Differences in resistance training regimens, such as intensity and duration, add to this variability and may affect the consistency of the observed effects. Potential publication bias also presents a limitation. Additionally, many studies used relatively short intervention periods, possibly underestimating the long-term benefits of resistance training. Finally, variability in patient characteristics, including age and OA severity, limits the generalizability of findings and highlights the need for further research to identify which subgroups might benefit most from resistance training.

To address the high variability in results, future studies should aim to standardize resistance training protocols, focusing on consistent variables such as intensity, frequency, duration, and specific exercises. Developing a standardized approach to resistance training could provide clearer insights into optimal protocols for improving function, reducing stiffness, and managing pain in knee OA patients. Another important area for future research is the need for longer-term studies. Many studies included in this meta-analysis utilized relatively short intervention periods, often fewer than 12 weeks. Given that muscle strengthening and functional improvements from resistance training can require sustained practice, longer-term studies are needed to assess the durability of resistance training benefits and its potential role in slowing disease progression. Extended follow-up periods would provide more comprehensive insights into the impact of resistance training on knee OA symptoms and patient quality of life over time. Additionally, more research is needed to explore how patient-specific factors, such as age, baseline physical function, and OA severity, influence the effectiveness of resistance training. Subgroup analyses within future studies could help identify whether certain populations are more likely to benefit from resistance training, facilitating a more personalized approach to OA management. Identifying patient characteristics associated with greater responsiveness to resistance training could help clinicians tailor interventions to individual needs more effectively.

## Conclusion

This systematic review and meta-analysis provide robust evidence supporting the role of resistance training as a valuable intervention for managing key outcomes in patients with knee osteoarthritis (OA). The findings demonstrate that resistance training significantly improves physical function and reduces stiffness, making it a promising non-pharmacological approach to enhancing mobility and daily functioning in this population. While there is some evidence to suggest a positive effect on pain reduction, the results were less consistent across pain measures, and the impact on quality of life remains inconclusive.

Despite its benefits, the study identified substantial variability in outcomes, underscoring the need for standardized training protocols to optimize effectiveness. The high heterogeneity observed across studies highlights the importance of individualizing resistance training regimens based on patient characteristics, such as age, baseline function, and disease severity. Additionally, the short duration of many included interventions suggests that longer-term studies are needed to fully understand the sustained benefits of resistance training and its potential to slow OA progression.

Clinicians are encouraged to incorporate resistance training into multimodal treatment plans for knee OA, particularly for improving function and stiffness. However, to address pain and quality-of-life concerns comprehensively, resistance training should be combined with other complementary therapies, such as aerobic or flexibility exercises and psychosocial interventions.

Future research should focus on standardizing resistance training protocols, conducting longer-term studies, and exploring personalized approaches to maximize therapeutic outcomes. Investigating the combined effects of resistance training with other interventions will also be crucial for addressing the multifaceted needs of patients with knee OA. Ultimately, this study reinforces the importance of resistance training as a cornerstone of conservative management for knee OA, with the potential to significantly improve patients’ lives when implemented effectively.

## Conflict of Interest

There is no conflict of interest.
